# Transcription Factor SmWRKY1 Positively Promotes the Biosynthesis of Tanshinones in *Salvia miltiorrhiza*

**DOI:** 10.3389/fpls.2018.00554

**Published:** 2018-04-27

**Authors:** Wenzhi Cao, Yao Wang, Min Shi, Xiaolong Hao, Weiwei Zhao, Yu Wang, Jie Ren, Guoyin Kai

**Affiliations:** ^1^Institute of Plant Biotechnology, Development Center of Plant Germplasm Resources, College of Life and Environment Sciences, Shanghai Normal University, Shanghai, China; ^2^College of Pharmacy, Zhejiang Chinese Medical University, Hangzhou, China

**Keywords:** *Salvia miltiorrhiza*, hairy roots, *SmWRKY1*, MEP pathway, tanshinones, metabolic engineering

## Abstract

Tanshinones, one group of bioactive diterpenes, were widely used in the treatment of cardiovascular diseases. WRKYs play important roles in plant metabolism, but their regulation mechanism in *Salvia miltiorrhiza* remains elusive. In this study, one *WRKY* transcription factor *SmWRKY1* was isolated and functionally characterized from *S. miltiorrhiza*. Multiple sequence alignment and phylogenetic tree analysis showed SmWRKY1 shared high homology with other plant WRKYs such as CrWRKY1. *SmWRKY1* was found predominantly expressed in leaves and stems, and was responsive to salicylic acid (SA), methyl jasmonate (MeJA), and nitric oxide (NO) treatment. Subcellular localization analysis found that SmWRKY1 was localized in the nucleus. Over-expression of *SmWRKY1* significantly elevated the transcripts of genes coding for enzymes in the MEP pathway especially 1-deoxy-D-xylulose-5-phosphate synthase (*SmDXS*) and 1-deoxy-D-xylulose-5-phosphate reductoisomerase (*SmDXR*), resulted in over fivefold increase in tanshinones production in transgenic lines (up to 13.7 mg/g DW) compared with the control lines. A dual-luciferase (Dual-LUC) assay showed that SmWRKY1 can positively regulate *SmDXR* expression by binding to its promoter. Our work revealed that SmWRKY1 participated in the regulation of tanshinones biosynthesis and acted as a positive regulator through activating *SmDXR* in the MEP pathway, thus provided a new insight to further explore the regulation mechanism of tanshinones biosynthesis.

## Introduction

*Salvia miltiorrhiza* Bunge, belonging to the *Lamiaceae* family, is a famous and traditional Chinese herbal plant that has been widely used for the treatment of cardiovascular and cerebrovascular diseases (Zhang et al., 2010; Kai et al., 2011). The abietane-type diterpenes in *S. miltiorrhiza* are the liposoluble tanshinones including dihydrotanshinone, tanshinone I, tanshinone IIA and cryptotanshinone with the same mother ring structure (Supplementary Figure S1), which exert a variety of biological activities such as antioxidant, heart-protection, antibacteria, and antitumor (Xu et al., 2010, 2015; Zhang et al., 2011; Chen et al., 2012; Gong et al., 2012). However, serious quality decrease and the low content of tanshinones in cultivated *S. miltiorrhiza* greatly limited the increasing market need (Hao et al., 2015; Zhou Y. et al., 2016). Therefore, it is important to improve the content of tanshinones by genetic engineering, which relies on deep understanding of the tanshinone biosynthetic pathway to *S. miltiorrhiza* (Liao et al., 2009; Zhou et al., 2016a, 2017).

Tanshinones represent a class of diterpenes the synthesis of which might depend on the plastidic 2-C-methyl-D-erythritol 4-phosphate (MEP) pathway, or on the cytosolic mevalonate (MVA) pathway that both built up the universal C5 precursors isopentenyl diphosphate (IPP) and its isomer dimethylallyl diphosphate (DMAPP) (Ge and Wu, 2005; Kai et al., 2011) (Supplementary Figure S2). 1-Deoxy-D-xylulose 5-phosphate synthase (DXS) catalyzes the condensation of pyruvate and glyceraldehyde 3-phosphate to yield 1-deoxy-D-5-phosphate (DXP), which is then converted to MEP by DXP reductoisomerase (DXR). DXS and DXR are frequently considered as analyzing the rate-limiting steps in the MEP pathway, and corresponding genes from *S. miltiorrhiza* have been cloned by our group (Liao et al., 2009; Yan et al., 2009). Subsequently, the C_20_ precursor geranylgeranyl diphosphate is synthesized by geranylgeranyl diphosphate synthase (GGPPS) (Kai et al., 2010). Finally, diverse diterpenoids are synthesized through terpene synthases/cylases including copalyl diphosphate synthase (CPS), kaurene synthase-like (KSL), miltiradiene oxidase CYP76AH1 and other modifying enzymes (Gao et al., 2009; Guo et al., 2013; Hao et al., 2015). The isolation and characterization of the above-mentioned tanshinone biosynthetic genes made it possible to improve tanshinone production at higher concentrations in *S. miltiorrhiza* through genetic manipulation (Kai et al., 2011; Shi et al., 2014, 2016a). Currently, some downstream steps of tanshinone formation still remain unknown for the moment, which limits metabolic engineering (Zhou et al., 2017).

Transcription factors (TFs) that are often capable of coordinately regulating multiple biosynthetic pathway genes, showed great promise to improve the production of active compounds by metabolic engineering. Therefore, molecular dissection of the regulatory mechanism of tanshinones biosynthesis may provide clues for activating the pathway and for producing tanshinones of high yield. Recently, heterologous expression of maize TF C1 in *S. miltiorrhiza* hairy roots elevated the accumulation of tanshinones through activating mevalonate 5-diphosphate decarboxylase (*SmMDC*) gene as the target (Zhao et al., 2015). The two kinds of TFs SmMYC2 and SmMYB9b have been isolated and functionally identified involved in the regulation of tanshinone biosynthesis (Zhou Y. et al., 2016; Zhang et al., 2017). However, less is known about WRKY TFs and their regulation mechanism in *S. miltiorrhiza* (Li et al., 2015).

WRKY TFs are one of the largest gene families unique to plants, which are involved in responses to biotic and abiotic stress and plant secondary metabolism (Suttipanta et al., 2011; Phukan et al., 2016). The significant feature of the WRKY TF is their WRKY domain which is approximately 60-amino acids long with the highly conserved amino acid sequence WRKYGQK being located at the N-terminal, and a non-typical zinc-finger-like motif C2HC (C–X_7_–C–X_23_–H–X_1_–C) or C2H2(C–X_4-5_–C–X_22-23_–H–X_1_–H) at the C-terminus (Xu et al., 2004; Lu et al., 2015). WRKY proteins can bind to the W-box *cis*-elements (T)TGAC(C/T) in the promoter region of some defense-related genes (Xu et al., 2004; Rushton et al., 2010; Liu et al., 2016). WRKY TFs can be separated into three sub-groups in accordance with the number of specific WRKY domains and zinc-finger-like motifs: Group I contains two WRKY domains and one C2H2 motif, Groups II has one WRKY domain and a C2H2 motif and Group III possesses one WRKY domain and a C2HC motif (Eulgem et al., 2000; Rushton et al., 2010). WRKYs have been found as being implicated in multiple physiological activities, including stress resistance, trichome development and secondary metabolism (Ma et al., 2009; Jiang et al., 2016; Liu et al., 2017). For example, *Gossypium arboreum* WRKY1 (GaWRKY1) was found to participate in the regulation of sesquiterpene biosynthesis in cotton by regulating the target gene (+)Δ-cadinene synthase (*CAD1*) (Xu et al., 2004). WRKY27, being responsive to various abiotic stresses interacted with GmMYB174, and then cooperatively inhibited *GmNAC29* expression, facilitating stress-tolerance of drought and cold in soybean (Wang et al., 2015). Recently, genes coding for WRKY have been isolated from some medicinal plants including *Catharanthus roseus* (Suttipanta et al., 2011), *Artemisia annua* (Chen et al., 2017), and *Withania somnifera* (Singh et al., 2017). Overexpression of *C. roseus WRKY1* (*CrWRKY1*) led to an up to threefold increase in serpentine levels in engineered hairy roots through binding to the W-box elements of the promoter tryptophan decarboxylase (TDC) involved in the indole alkaloid (TIA) biosynthetic pathway (Suttipanta et al., 2011). The WRKY TF GLANDULAR TRICHOME-SPECIFIC WRKY 1 (AaGSW1) positively regulated the expression of *AaCYP71AV1* and *AaORA* by conjunction to the W-box motifs of the promoters (Chen et al., 2017). A WRKY TF from *W. somnifera* bound to the W-box region in the promoters of squalene synthase and squalene epoxidase, regulating the accumulation of triterpenoids in *W. somnifera* including phytosterols and withanolides (Singh et al., 2017). However, functional WRKYs related to secondary metabolism of tanshinones or salvianolic acids in *S. miltiorrhiza* have not been reported.

In this study, the gene *SmWRKY1* coding for a WRKY TF (named as *SmWRKY1*) has been isolated and functionally characterized. Phylogenetic analysis showed that SmWRKY1 shared high homology with AtWRKY70, CrWRKY1, and GaWRKY1. Multiple sequence alignment revealed that the nucleus-localized SmWRKY1 contained one WRKY domain and a C2HC motif and could be classified into group III WRKY TFs. Introduction of *SmWRKY1* into *S. miltiorrhiza* hairy roots increased the transcripts of *SmDXS* and *SmDXR* involved in the MEP pathway, resulting in higher level of tanshinones in transgenic lines compared with the control lines (2.175 mg/g DW). The highest content of tanshinones was produced in *SmWRKY1-3* line at 13.731 mg/g DW, which was 5.3-fold higher than the control. A Dual-LUC assay revealed that SmWRKY1 activated the expression of *SmDXR* by directly binding to its promoter. Taken together, our work revealed that *SmWRKY1* positively elevated the accumulation of tanshinones, which provides a new insight to further excavate the regulation mechanism of tanshinones biosynthesis.

## Materials and Methods

### Plant Materials and Elicitor Treatments

*Salvia miltiorrhiza* seedlings were cultivated as we reported before (Kai et al., 2011; Shi et al., 2014). *N. benthamiana* were prepared according to previous reports (Shi et al., 2016b; Zhou Y. et al., 2016).

For elicitor induction treatments, methyl jasmonate (MeJA) and salicylic acid (SA) were prepared as before (Kai et al., 2012; Hao et al., 2015). For NO elicitation, firstly 100 mM [Na_2_]Fe(CN)_5_NO]x2 H_2_O (SNP) solution was obtained, and then applied to cultures to 100 μM. All the above-mentioned solutions were sterilized through 0.22 μm filters (Pall Corporation, United States). And solvent of the equivalent volume was added into the control group.

### Isolation and Bioinformatics Analysis of *SmWRKY1*

A local transcription database of *S. miltiorrhiza* was built up as reported previously (Shi et al., 2016b). One partial *WRKY* sequence with high homology to other plants *WRKY* genes, albeit lacking a part of 3′-terminal was chosen for further study. Gene-specific forward primer *SmWRKY1*-F605 was designed to amplify the 3′ end of *SmWRKY1* by combination with the reverse primer AUAP using rapid amplification of cDNA ends (RACE) as reported before (Liao et al., 2009; Kai et al., 2010; Zhang et al., 2011). 5′-sequence and 3′-terminal products was aligned and assembled to obtain the full-length cDNA sequence of the putative *SmWRKY1* gene. Primer pairs *SmWRKY1-KF* and *SmWRKY1-KR* were synthesized for amplification of the full ORF of *SmWRKY1* according to the procedure as described below: initial denaturation at 94°C for 10 min, 35 cycles of 94°C for 45 s, 55°C for 45 s and 72°C for 90 s, followed by a final extension at 72°C for 10 min. All primers used for identification of *SmWRKY1* were listed in Supplementary Table S1. The characteristics of *SmWRKY1* were further analyzed by a series of bioinformatics tools. Nucleotide blast, protein blast and ORF Finder were used to analyze nucleotide sequence and the complete open reading frame^1^. MEGA 6 was applied to construct a phylogenetic tree by the neighbor-joining (NJ) method and 1000 replications were performed for bootstrap values. Multiple sequences alignment between SmWRKY1 and other plant WRKYs were performed on amino acids level by using Clustal X with default parameters (Shi et al., 2016b; Zhou Y. et al., 2016).

### Expression Pattern of *SmWRKY1* in Different Tissues and Under Various Elicitors Treatments

Different tissues including taproot, stem, leaf, flower, and seed were gathered from 2-year-old *S. miltiorrhiza* plants in maturation. Elicitor treatments were conducted on *S. miltiorrhiza* hairy roots sub-cultured for 60 days infected with *Agrobacterium* C58C1. Hairy roots were harvested at selected time points (0, 0.5, 1, 2, and 4 h) after MJ treatment. And for SA and NO induction, hairy roots were collected at 0, 3, 4, 6, 9, and 12h after treatment. All the treated samples were immediately frozen in liquid nitrogen and stored for analyzing the expression profiles of *SmWRKY1*.

### Subcellular Localization of SmWRKY1

To analyze the subcellular localization of SmWRKY1, PCR products of *SmWRKY1* ORF with *Bgl*II and *Kpn*I restriction sites were digested with *Bgl*II and *Kpn*I and cloned into the vector *pMON530* to generate the vector *pMON530-SmWRKY1-GFP*. The constructed expression vector was transferred into *Agrobacterium* strain ASE and injected into 40-day-old tobacco leaves. GFP fluorescence was observed after 48 h cultivation using the confocal microscope (Carl Zeiss) (Shi et al., 2016b; Zhou et al., 2016a).

### Generation of Transgenic *SmWRKY1* Hairy Roots

The full-length coding sequence of *SmWRKY1* with restriction sites *Spe*I and *BstE*II was cloned and inserted into modified *pCAMBIA2300^sm^* vector (having replaced the small fragment digested by *Eco*RI and *Hind*III with the corresponding *GFP-GUSA* gene expression cassette from *pCAMBIA1304*) under the control of the CaMV 35S promoter to generate *pCAMBIA2300^sm^-SmWRKY1*. *Agrobacterium rhizogenes* strain C58C1 containing *pCAMBIA2300^sm^-SmWRKY1* was used to infect the aseptic explants and the empty *pCAMBIA2300^sm^* was regarded as the control. The transformation procedure was the same as in our previous study (Kai et al., 2011; Shi et al., 2014, 2016a,b; Zhou Y. et al., 2016). Hairy roots in good state were sub-cultured and primer pairs *CaMV35S*-F23 and *SmWRKY1-*QR were used to identify positive colonies by polymerase chain reaction (PCR) analysis. Genomic DNA was isolated from individual hairy root sample by the cetyltrimethyl ammonium bromide method as previously reported (Zhou et al., 2016a,b). Identified positive-colonies were cut into segments of 4 cm for further shake-flask culture in 100 ml 1/2 MS medium and cultured at 25°C on an orbital shaker shaking at the speed of 100 rpm in darkness (Shi et al., 2016a,b). Primer sequences were listed in Supplementary Table S1.

### Total RNA Isolation and Relative Expression Analysis via qRT-PCR

Expression profiles of *SmWRKY1* and several key enzyme genes involved in tanshinones biosynthetic pathway were investigated by real-time quantitative PCR analysis (qRT-PCR). Total RNA for reverse transcription (RT) reaction was extracted as described before (Shi et al., 2016b). qRT-PCR was carried out by gene-specific primers (listed in Supplementary Table S1) using previously reported procedure (Shi et al., 2016b).

### Dual-Luciferase (Dual-LUC) Assay

For the dual-luciferase (Dual-LUC) assay, the promoters of *SmDXR* and *SmDXS2* with *KpnI* and *XhoI* restriction sites were cloned into *pGREEN 0800* to drive the luciferase reporters, respectively. The complete ORF of *SmWRKY1* was inserted into the *pCAMBIA2300^sm^* vector as effector. The *pCAMBIA2300^sm^-SmWRKY1* and *pCAMBIA2300^sm^* empty plasmid were transferred into *A. tumefaciens* strain GV3101 individually. The *pGREEN-pSmDXR, pGREEN-pSmDXS2* was each co-transformed with the helper plasmid pSoup19 into GV3101, and the assay was conducted as described before (Zhang et al., 2015). The *pCAMBIA2300^sm^* empty plasmid was used as a negative control. The 35S promoter-driven gene for *Renilla* (*reniformis*) luciferase was taken as an internal control. Each measurement was repeated for three biological times. The reporter and the effector strains were mixed in a ratio of 1:1, followed by injection of tobacco leaves. Two days after injection the leaf samples were collected for the dual-LUC assay following the manufacturer’s (Promega) protocol.

### Tanshinones Analysis

The 60-day-old hairy roots were dried at 50°C to constant weight in an oven. Approximate 200 mg dried hairy roots were ground into powder and immersed in 16 mL methanol/dichloromethane (3:1, v/v) for extraction of tanshinones as described earlier (Hao et al., 2015). HPLC analysis was performed by using an Agilent 1260 apparatus equipped with a Waters reversed-phase C18 symmetry column, and the detection conditions were as described previously (Shi et al., 2016b).

### Statistical Analysis

All experiments including identification of positive hairy roots lines, qRT-PCR, HPLC analysis of tanshinones, Dual-LUC assay were performed three times and the results are shown as means values of three independent repeats ± standard deviation. Statistical significance was determined by the one sample *t*-test and one-way analysis of variance using SPSS 11.5 software (SPSS).

## Results

### Isolation and Bioinformatics Analysis of *SmWRKY1*

By searching our local transcriptome database, a *WKRY* fragment with 5′ untranslated region (UTR) but lacking a part of 3′ terminal sequence was chosen for further research because it showed high homology with *GaWRKY1* and *CrWRKY1* as well as *Arabidopsis thaliana WKRY70*. By 3′-RACE, an about 432 bp PCR products was obtained in which a 3′-UTR of 238 bp was found downstream from the stop codon. After sequence assembly, the full-length gene was cloned and designated as *SmWRKY1*. Its sequence consists of 17 bp 5′UTR, a complete 789 bp ORF, encoding a 262 amino acid protein, along with 238 bp 3′ UTR.

**FIGURE 1:**
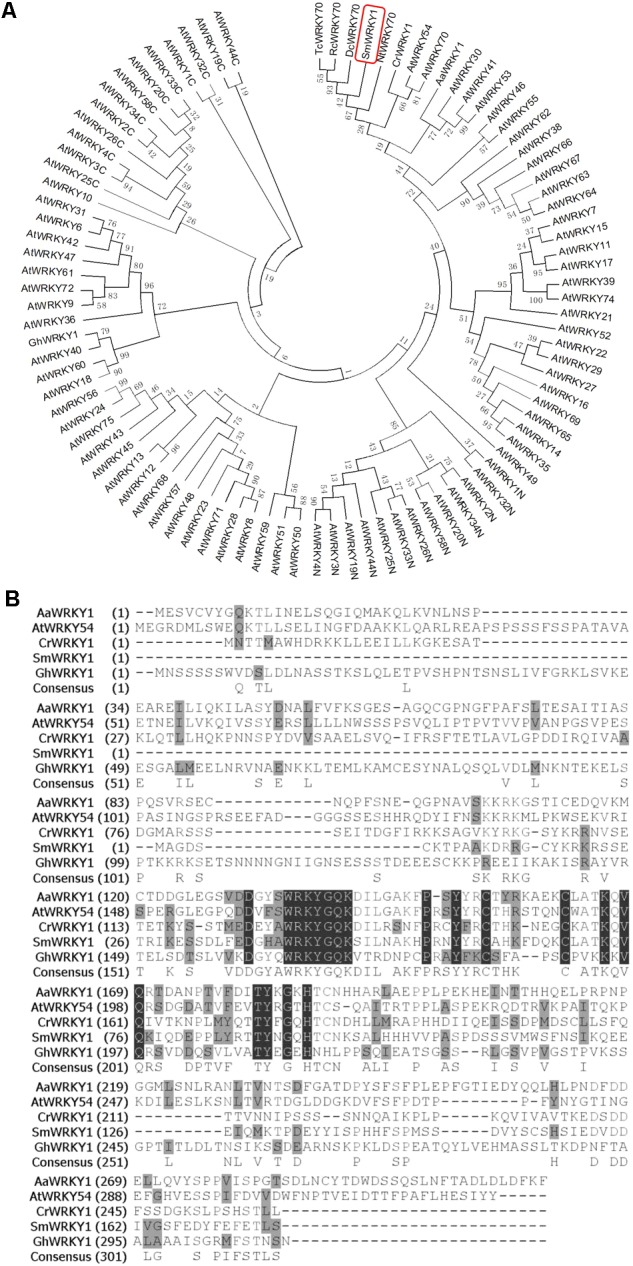
**(A)** Multiple alignment of *SmWRKY1* with related WRKY proteins from other plant species. Black boxes indicate identical residues; gray boxes indicate identical residues for at least three of the sequences. **(B)** Phylogenic tree analysis of SmWRKY1 and WRKY TFs from *Arabidopsis thaliana, Artemisia annua, Catharanthus roseus, Nicotiana tabacum*, etc. Phylogenic tree was constructed on MEGA6.0 by using neighbor-joining method and the bootstrap values were obtained for 1000 replications.

To further figure out the biological characteristics and phylogenetic relationship of *SmWRKY1*, a series of bioinformatic analyses were performed. Multiple alignment of SmWRKY1 with related WRKY proteins from other plant species revealed that SmWRKY1, AaWRKY1, and CrWRKY1 all contained a conserved WRKY domain (WRKYGQK) and a special zinc-finger like motif in its C-terminal, which is typical of the group III of WRKY TFs (**Figure 1A**), indicative of their similar function. The amino acid sequence alignment of SmWRKY1 with (other) plant WRKYs allowed for constructing a neighbor-joining tree (**Figure 1B**). The results revealed that SmWRKY1 shared 62, 49, 37, and 29% identities with EgWRKY70, NtWRKY70, CrWRKY1, and AaWRKY1, respectively.

### Tissue and Induction Expression Profiles of *SmWRKY1*

**FIGURE 2:**
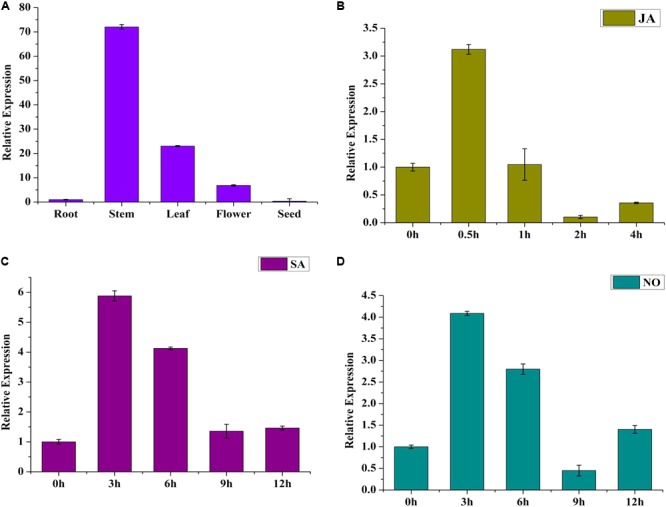
**(A)** Expression pattern of *SmWRKY1* in different tissues. Each tissue was obtained from several individual 2-year-old *S. miltiorrhiza* plants in nature. Transcript abundance of *SmWRKY1* is normalized to actin by the method of 2^-ΔΔ^*^C^*^t^. **(B)** Time course of the expression level of *SmWRKY1* after SA treatment as determined by qRT-PCR. **(C)** The expression level of *SmWRKY1* after SA treatment for selected time points measured by qRT-PCR. **(D)** The expression level of *SmWRKY1* after NO treatment for selected points by qRT-PCR analysis, respectively. Values are means ± standard deviation of triplicate analyses.

To investigate the tissue expression pattern of *SmWRKY1*, roots, stems, leaves, flowers, and seeds from 2-year-old *S. miltiorrhiza* plants were analyzed. *SmWRKY1* showed high expression in leaves and stems, low expression in flowers and roots, and its transcript was barely detected in seeds (**Figure 2A**). This result indicated that *SmWRKY1* is not constitutively expressed in tissue.

To study whether *SmWRKY1* could respond to exogenous hormone treatment, 60-day-old *S. miltiorrhiza* hairy roots were treated with MeJA for different time points, where that at 0 h was used as control and expression was detected by qRT-PCR. The result indicated that exogenous MeJA induced *SmWRKY1* expression (**Figure 2B**), reaching a peak at 0.5 h, arising to about threefold. Thereafter, the transcript level declined rapidly within 2 h. Treatment with SA and NO did also induce *SmWRKY1* expression, with a maximum peak being reached at 3 h, followed by a gradual decrease over 12 h (**Figures 2C,D**).

### Subcellular Localization of *SmWRKY1*

**FIGURE 3:**
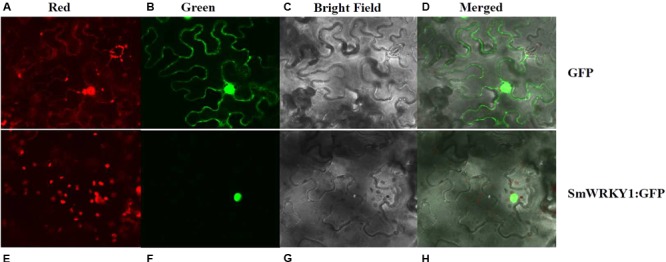
Subcellular localization of SmWRKY1. **(A–D)** The free GFP expressed in *N. benthamiana* leaves. **(E–H)** SmWRKY1:: GFP expressed in *N. benthamiana* leaves.

To experimentally confirm the subcellular localization of SmWRKY1, *SmWRKY1* was cloned into the *pMON530* vector to fuse with green fluorescent protein (GFP) reporter gene to generate vector *pMON530-SmWRKY1-GFP*. Then, the constructed vector and the *pMON530* (used as the control) was transformed into the *ASE* strain and expressed in tobacco leaves, respectively. In the leaves of control vector transformed plant, the fluorescence of GFP was detected in the cytoplasm and nucleus (**Figure 3**). In contrast (to this), the fluorescence of SmWRKY1/GFP fusion protein was only found in nucleus. The expression pattern was consistent with the character of SmWRKY1 as a TF.

### Acquisition of *SmWRKY1* Transgenic Hairy Roots

**FIGURE 4:**
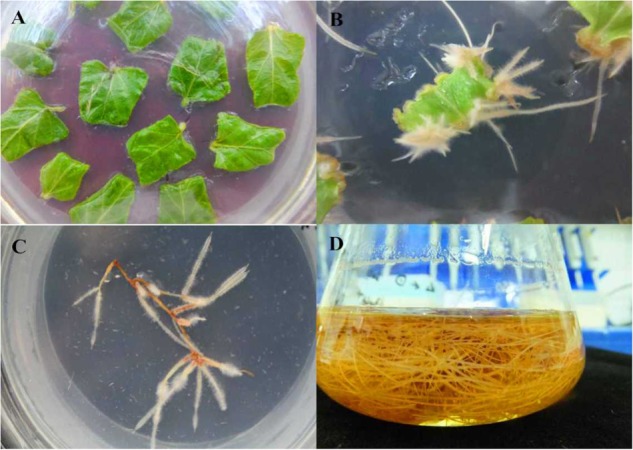
Generation of transgenic hairy root of *S. miltiorrhiza.* **(A)** *S. miltiorrhiza* explants on ½ MS medium. **(B)** The growing hairy root on the infected *S. miltiorrhiza* explants. **(C)** Monoclone of hairy root. **(D)** Hairy roots culture in ½ MS medium.

**FIGURE 5:**
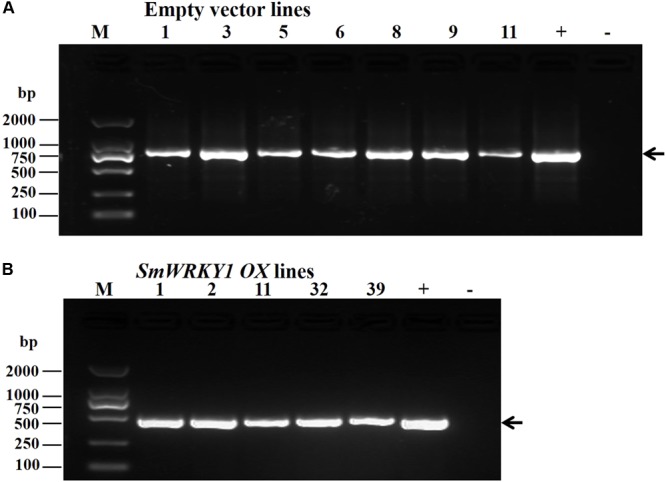
**(A)** Identification of positive transgenic hairy root lines by PCR. (*GusA-*F and *GusA-*R were used to identify empty vector *pCAMBIA2300*^sm^ transformed lines **(B)** Primers *CaMV35S*-F23 and *SmWRKY1-*QR were used to identify the positive colony of *SmWRKY1* overexpression transgenic lines).

**FIGURE 6:**
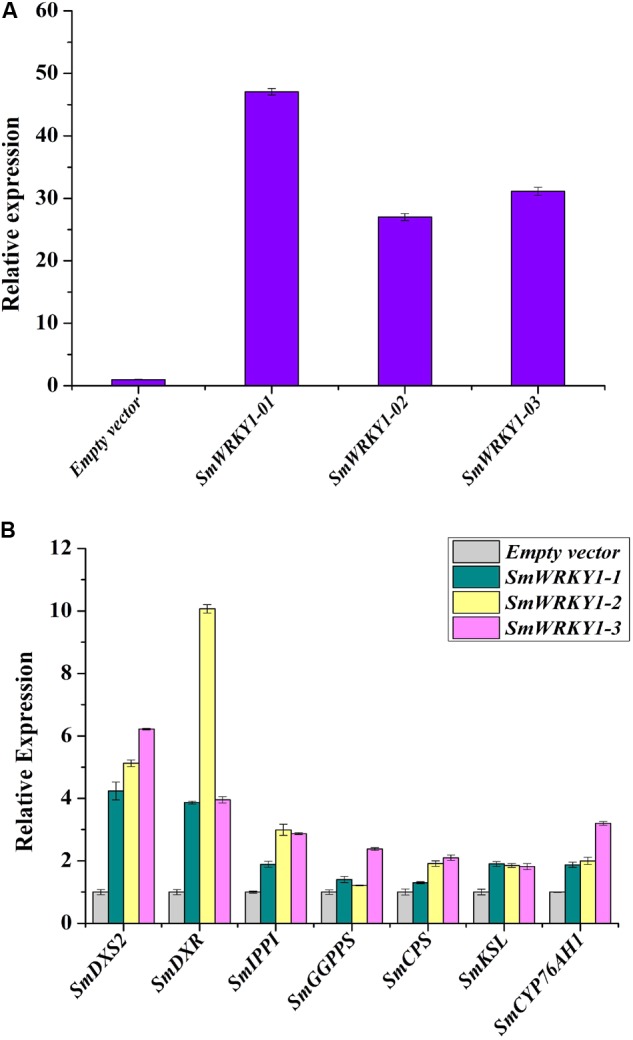
Transcript levels of *SmWRKY1* and genes related to tanshinones biosynthesis in *SmWRKY1* transgenic hairy roots. Expression of *SmWRKY1* was analyzed by qRT-PCR. All data are means of three replicates, with error bars indicating Standard Deviation. **(A)** The transcript level of *SmWRKY1* in *SmWRKY1* transgenic hairy roots. **(B)** The transcript level of the key tanshinones biosynthesis genes in *SmWRKY1* transgenic hairy roots.

To further investigate the function of *SmWRKY1* in *S. miltiorrhiza*, we inserted *SmWRKY1* into a modified *pCAMBIA2300*^sm^ vector (Supplementary Figure S4). Then the recombinant overexpression vector *pCAMBIA2300*^sm^*-SmWRKY1* was introduced into *A. rhizogenes* strain C58C1 and used to infect *S. miltiorrhiza* explants, and the empty vector *pCAMBIA2300*^sm^ was used as control. After 2–3 weeks the fresh hairy roots differentiated from the stem and leaf explant as shown in **Figure 4**. The positive lines carrying the *SmWRKY1* gene were verified by PCR. The positive rate was 20.5% among the 39 samples (**Figure 5**). qRT-PCR analysis of the expression of *SmWRKY1* in over-expression lines found that *SmWRKY1* expressed 20–48 times higher than in the empty vector control transformed lines (**Figure 6A**). The three high expression lines including 1, 2, and 32 (designated as 3) were chosen for further analysis.

### Expression of Tanshinone Biosynthetic Genes in *SmWRKY1* Transgenic Hairy Roots

To study whether *SmWRKY1* participated in the regulation of tanshinone biosynthesis, transcript levels of several genes related to tanshinones biosynthesis in *SmWRKY1* transgenic hairy root were analyzed by qRT-PCR. Several tanshinone biosynthesis pathway genes were up-regulated in the *SmWRKY1*-overexpressing hairy roots (**Figure 6B**), the most striking ones were *SmDXS2* and *SmDXR* genes, which increased 4–6-fold and 4–10-fold as compared with the control, respectively. Though the expression of *SmIPPI, SmGGPPS, SmCPS, SmKSL*, and *SmCYP76AH1* was a little lower than that of *SmDXS* and *SmDXR*, their expression in overexpression lines was two–fourfold higher than in the control lines. In contrast, the expression of all these seven tanshinones biosynthesis pathway genes was significantly decreased in the knock-down lines. All these results suggested that *SmWRKY1* may be a positive regulator in tanshinones biosynthesis.

### SmWRKY1 Activates the Transcription of *SmDXR in Vivo*

**FIGURE 7:**
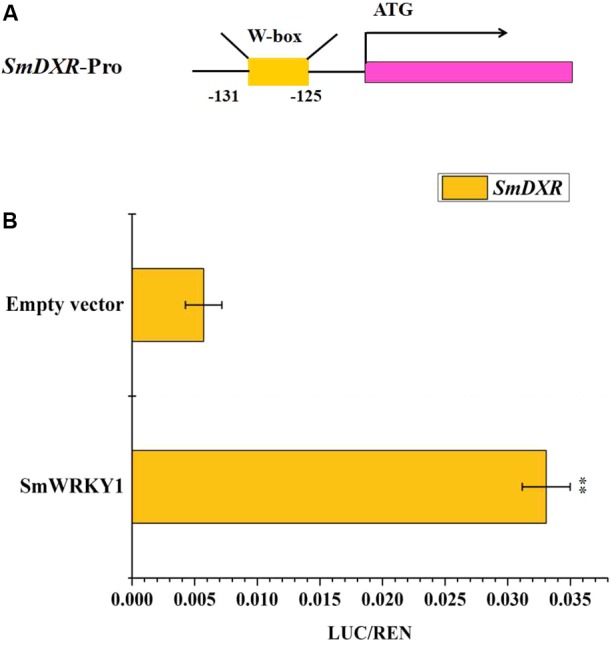
The *SmDXR* promoter was fused to the luciferase (LUC) reporter and the promoter activity was determined by a transient dual-LUC assay in tobacco. The value of LUC activity/Renilla (REN) luciferase was regarded as the activating activity. Error bars indicate SD (*n* = 3). Student’s *t*-test: ^∗^*P* < 0.05, ^∗∗^*P* < 0.01. **(A)** The analysis of motifs in *SmDXR* promoter. **(B)** The Dual-luciferase assay that *SmWRKY1* activates the promoter of *SmDXR*.

Expression profiles showed that *SmWRKY1* significantly promotes the expression of *SmDXR* and *SmDXS2*, coding for enzymes being in charge of pivotal catalytic steps of tanshinone accumulation. By analyzing the sequence of *SmDXR* and *SmDXS2* promoters, we found a W-box in the promoter of *SmDXR* (**Figure 7A**). Then the dual luciferase (Dual-LUC) method was employed to verify whether SmWRKY1 protein activates the transcription of *SmDXR* and *SmDXS2* or not. The results showed that *SmWRKY1* over-expression led to an increase by 6 fold of *SmDXR* expression (**Figure 7B**).

### Accumulation of Tanshinone Was Obviously Affected by *SmWRKY1*

**FIGURE 8:**
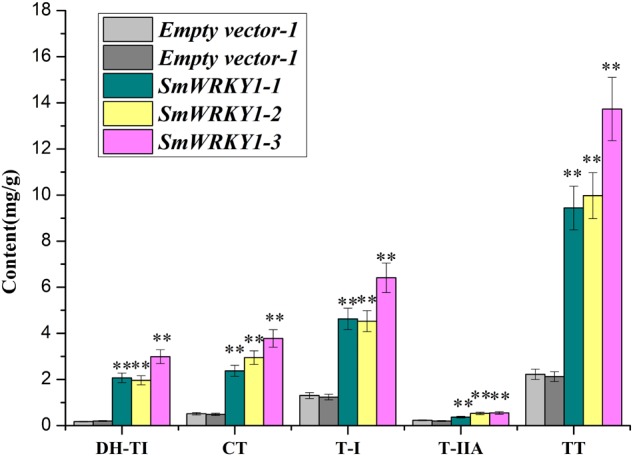
The production of tanshinone in *SmWRKY1* transgenic hairy roots compared with control tissue, detected by HPLC. All data are means of three replicates, with error bars indicating Standard Deviation. ^∗^, significant at *P* < 0.05, ^∗∗^, highly significant at *P* < 0.01.

Based on the quantitative data, we wanted to further evaluate whether the expression of *SmWRKY1* in transgenic hairy roots affects the content of tanshinone. Three overexpression lines and two knock-down lines were used to examine four monomers of tanshinones, including cryptotanshinone, dihydrotanshinone I, tanshinone I, tanshinone IIA in hairy roots by HPLC (Supplementary Figure S3). The results showed that the content of cryptotanshinone (2.4–3.8 mg/g DW), dihydrotanshinone I (2.0–3.0 mg/g DW), tanshinone I (4.5–6.4 mg/g DW), and tanshinone IIA (0.4–0.6 mg/g DW) were significantly up-regulated and the total tanshinone had risen to 9.4–13.7 mg/g DW in overexpression lines. Among them *pCAMBIA2300^sm^*-*SmWRKY1*-3 lines accumulated the highest content of total tanshinone, which was 6.3 times of the control (**Figure 8**). These results further confirmed the positive role of *SmWRKY1* in the regulation of tanshinone biosynthesis.

## Discussion

The regulation of isoprenoid biosynthesis in plants has been in the focus of many laboratories around the world starting latest in about 1950. The plant *S. miltiorrhiza* is of some medical interest due to the presence of various diterpenoids of the tanshinone type. Thus, genetic engineering has become an effective and important way to increase the accumulation of active ingredients in *S. miltiorrhiza*. In the past decade, much progress has been made in isolation of tanshinone biosynthetic genes and metabolic regulation of tanshinone biosynthesis by expression of single or multiple target genes with various combination (Kai et al., 2011; Shi et al., 2014, 2016a). Overexpression of *SmDXS* in transgenic hairy root lines can significantly enhance the production of tanshinones (Zhou Y. et al., 2016). In the meantime *SmDXR* also been identified as an important enzyme gene in tanshinone biosynthesis as its over-expression led to significantly improve the production of tanshinones in hairy root lines (Shi et al., 2014). Co-expression of *SmHMGR* and *SmGGPPS* increased tanshinone production significantly in a transgenic *S. miltiorrhiza* hairy root line HG9 (Kai et al., 2011). In addition, introduction of *SmHMGR* and *SmDXR* into *S. miltiorrhiza* hairy roots enhanced the content of tanshinones to 3.25 mg/g DW (Shi et al., 2014). Simultaneous introduction of *SmGGPPS* and *SmDXSII* into *S. miltiorrhiza* hairy root significantly improved the production of tanshinones and increased production of carotenoids, gibberellins and chlorophyll in *A. thaliana* plants (Shi et al., 2016a). However, tanshinones engineering may be impeded by the fact that the multiple steps in the downstream part of tanshinone biosynthesis are only partially characterized, although the identification of those enzymes is ongoing. Therefore, the attempts undertaken were not always crowned by a big success due to the huge and complex regulatory network.

Currently, people began to realize that it would be more effective to use TFs that might activate whole pathways, instead of individually over-expressing genes that encode putatively rate-limiting enzymes. TFs are divided into different families in plants, such as bHLH, MYB, ERF, WRKY, and so on. Each of them has their specific functions in the regulation of secondary metabolism of plants. Recently, Zhang et al. (2017) reported that overexpression of *SmMYB9b* enhances tanshinone concentration in *S. miltiorrhiza* hairy roots (2.2-fold improvement over the control), but the direct target gene in tanshinones biosynthetic pathway was not identified. In addition, Zhou Y. et al. (2016) reported on ectopic RNA interference (RNAi)-mediated knockdown experiments, which suggested that the accumulation was impaired by the loss of function in *SmMYC2a/b*. However, the opposite effect by OE of *SmMYC2a/b* had not been studied yet, and the identification of direct target genes in tanshinone biosynthesis is also awaiting. In addition, to our knowledge, no WRKY TF from *S. miltiorrhiza* has been functionally characterized yet to date. In present study, we functionally identified a new TF SmWRKY1 from *S. miltiorrhiza* and found that OE of *SmWRKY1* can obviously promote the accumulation of tanshinones in transgenic lines. The highest tanshinone production was observed in transgenic SmWRKY1-3 with the concentration of 13.7 mg/g DW (5.3-fold improvement than the control), to our knowledge, this is the highest tanshinone content achieved through genetic engineering.

Meanwhile, expression profile analysis showed that overexpression of *SmWRKY1* can promote the transcripts level of several biosynthetic genes especially *SmDXR*, and previous studies have proved that WRKY TFs could directly bind to the W-box of related genes from different signal pathways (Eulgem et al., 2000). Thus we scanned the promoter of *SmDXR*, and a W-box motif was found, which implied that it can be bound and regulated by SmWRKY1. Thereafter, the Dual-LUC assay proved that SmWRKY1 can transcriptionally activate *SmDXR* expression by directly binding to its promoter region, in good agreement with that *SmDXR* has been identified an important target gene in tanshinone biosynthesis (Shi et al., 2014). In some other medicinal plants including *C. roseus* (Suttipanta et al., 2011), *A. annua* (Chen et al., 2017), and *W. somnifera* (Singh et al., 2017), WRKYs have also been found to activate the transcripts of different target genes by binding to the W-box in their promoters, suggesting that the W-box was their recognition *cis*-element.

## Conclusion

Our work functionally identified a new TF *SmWRKY1* gene from *S. miltiorrhiza* and found that overexpression of *SmWRKY1* can obviously promote the accumulation of tanshinone in transgenic lines. The Dual-LUC assay indicated that SmWRKY1 positively regulated tanshinone biosynthesis through a direct target gene *SmDXR* involved in the MEP pathway. Our study may provide a new insight by genetic engineering strategy with functional TFs to improve the yield of target compounds in *S. miltiorrhiza* and also a certain basis to analyze the entire regulation network of tanshinone biosynthesis.

## Author Contributions

GK conceived and designed the study. WC, YaW, MS, XH, and WZ performed the experiments and analyzed the data. WC, MS, JR, and YuW drafted the manuscript, and GK revised the manuscript.

## Conflict of Interest Statement

The authors declare that the research was conducted in the absence of any commercial or financial relationships that could be construed as a potential conflict of interest.

## Footnotes

1 http://www.ncbi.nlm.nih.gov
