# Supplementary material for: Transcription Factor SmWRKY1 Positively Promotes the Biosynthesis of Tanshinones in Salvia miltiorrhiza
Source: Front Plant Sci. 2018 Apr 27;9:554. doi: 10.3389/fpls.2018.00554 (PMC5934499; doi:10.3389/fpls.2018.00554)
Supplement: Supplementary file 1 [file Data_Sheet_1.docx]

| Primers | Sequences (5′-3') | |  |
| --- | --- | --- | --- |
| *SmWRKY1-LIKE1*-F605 | | GGAGCTTCAATTCGATCAAACAGG | |
| *SmWRKY1*-KF | | ACTAGTATGGCATCTTCCTCCTGCGTTCAC | |
| *SmWRKY1*-KR | | GGTCACCTCAACTTAAGGTTTCAAATTCG | |
| *SmWRKY1*-520QF | | ACCTACAACGGCCAACACACT | |
| *SmWRKY1*-659QR | | TCGTCCGGTGTTTTCATTTG | |
| *SmActin*-1011F | | AGCACCGAGCAGCATGAAGATT | |
| *SmActin*-1210R | | AGCAAAGCAGCGAACGAAGAGT | |
| *SmDXS*-1828F | | TTGGAGATTGGGAAGGGAAGGAT | |
| *SmDXS-*1980R | | AGGCTTGCAGAATCTCGCATCAG | |
| *SmDXR*-1248F | | CGACGAGAAAATCGGATACCTGG | |
| *SmDXR*-1424R | | CATACAAGAGCAGGACTCGAACCG | |
| *SmIPPI*-1422F | | GCAACGATCCACAACTAAGGT | |
| *SmIPPI*-1572R | | ATGCCGAGTTCATCCAACAG | |
| *SmGGPPS*-603F | | GCTGTGCTCGCAGGGGATG | |
| *SmGGPPS*-774R | | ATCGCCGGTGCAGTTCAGG | |
| *SmCPS*-459F | | GATCGCCTCGTCAATACCAT | |
| *SmCPS-*609R | | TTCGAACCCACAAGTCATGT | |
| *SmKSL*-1480F | | GTGTGACCCTTCTGCTAGCA | |
| *SmKSL*-1630R | | TGCATTGTCTTGGGAAGATG | |
| *SmCYP76AH1*-1010F | | TCGTGGATGAGTCGGCAAT | |
| *SmCYP76AH1*-1168R | | TGAGTATCTGAGTTCCCT | |
| 0800-Sm*DXS2*-Pro-KpnI-F-1949 | | GGGGTACCATGAGCCGGTCCTTTACTTCTGTTC | |
| 0800-Sm*DXS2*-Pro-XhoI-R-17 | | CCCTCGAGTCGACCCAAGGGGAAGTGAAGC | |
| 0800-Sm*DXR*-Pro-KpnI-F-1974 | | GGGGTACCAAAGGGCATTTTAGGGAGGGAGAGG | |
| 0800-Sm*DXR*-Pro-XhoI-R； | | CCCTCGAGGGCTTATCCACGCTCGAATGCACA | |

**Table S1** All primers used in this study*.*


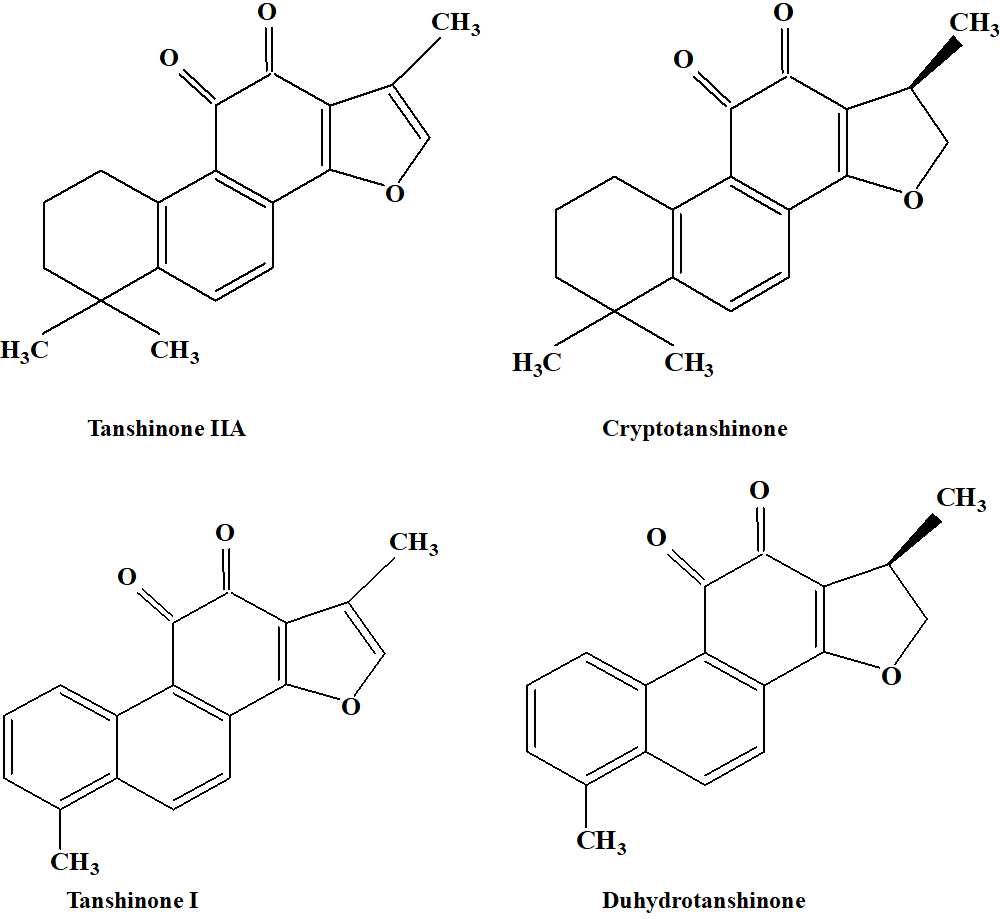


**Fig. S1.** The Chemical structural formula of tanshinone compounds

**
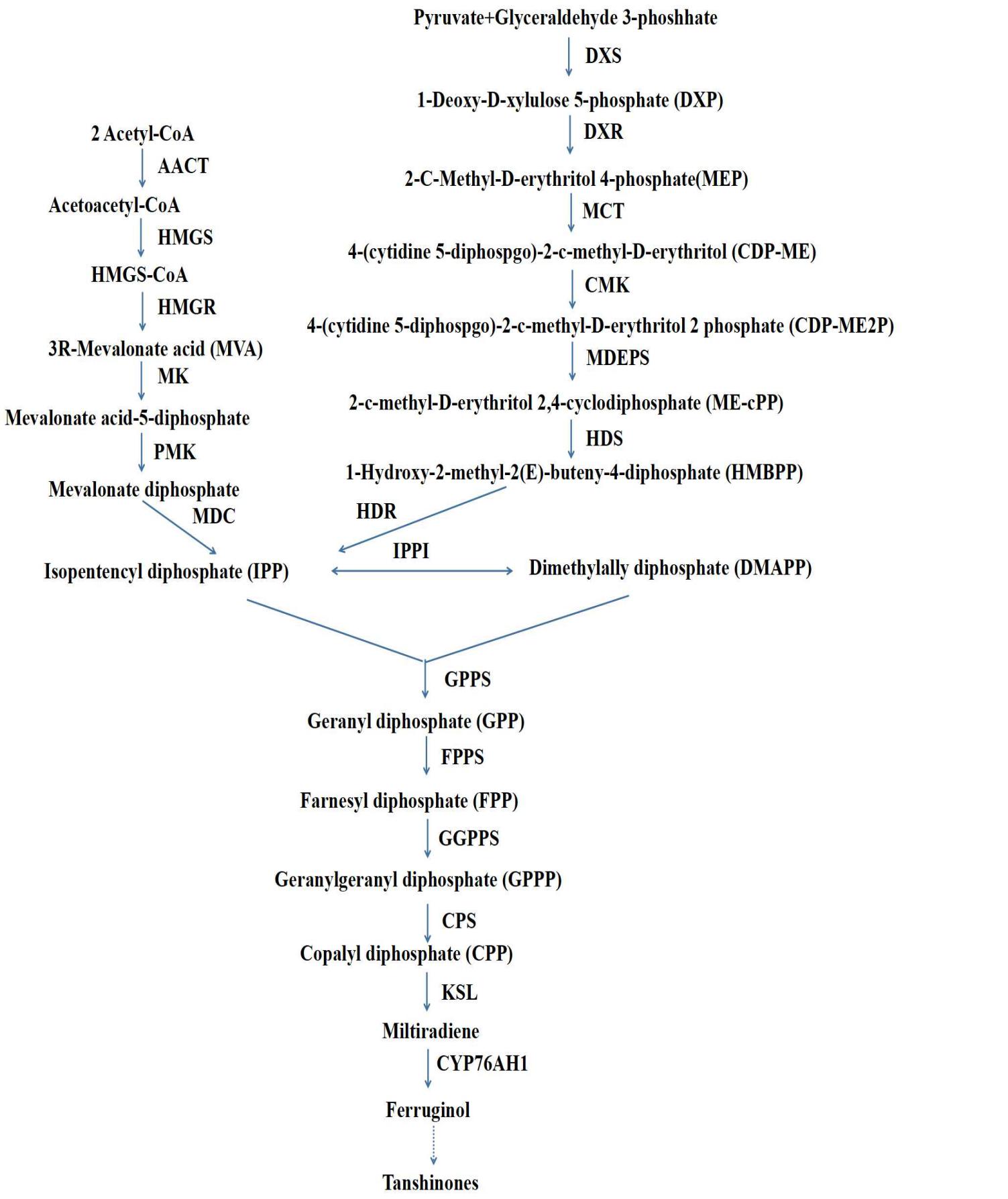
**

**Fig. S2.** The biosynthesis pathway of tanshinones

**Cryptotanshinone**

**Standards**


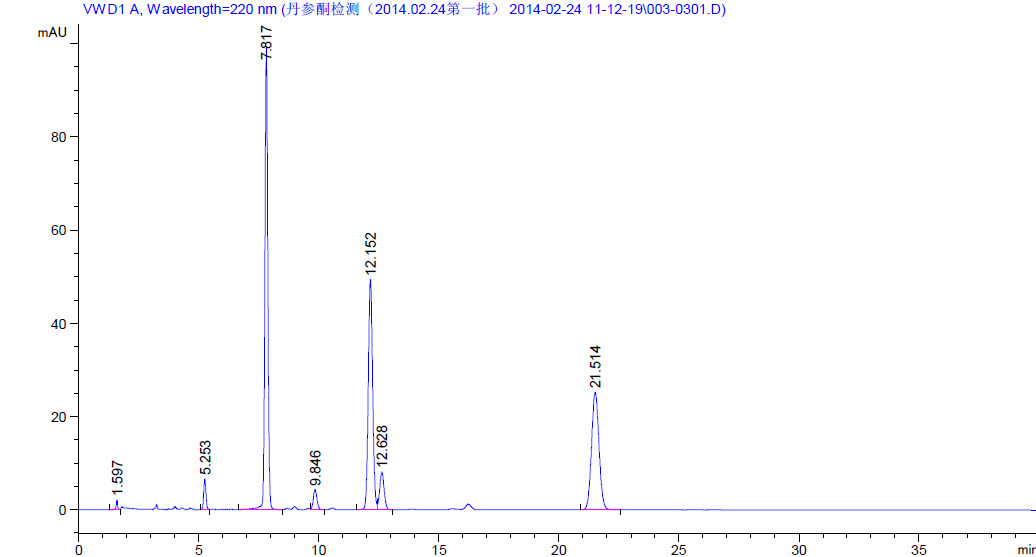


**Duhydrotanshinone**

**Tanshinone IIA**

**Tanshinone I**


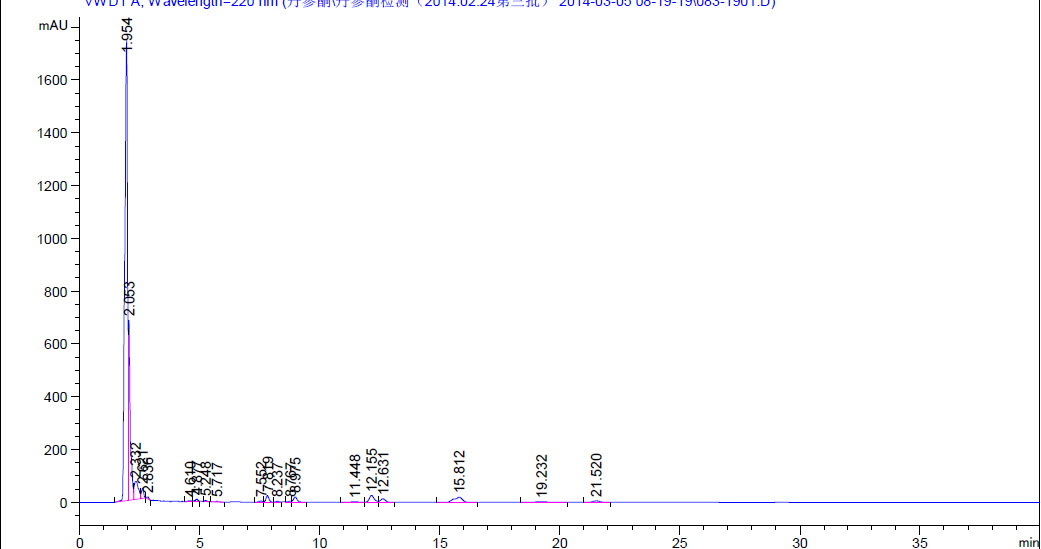


**Empty vector-1**


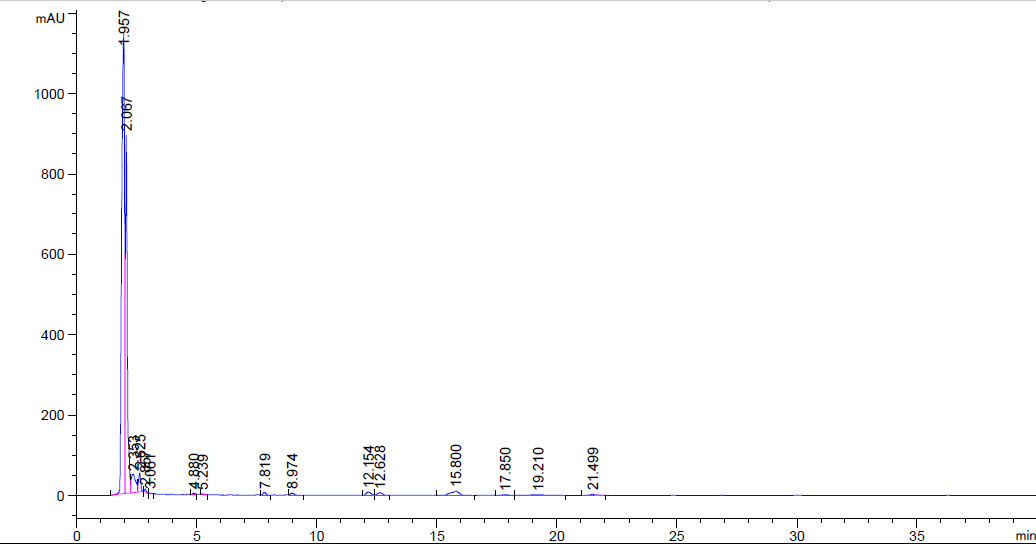


**Empty vector-2**


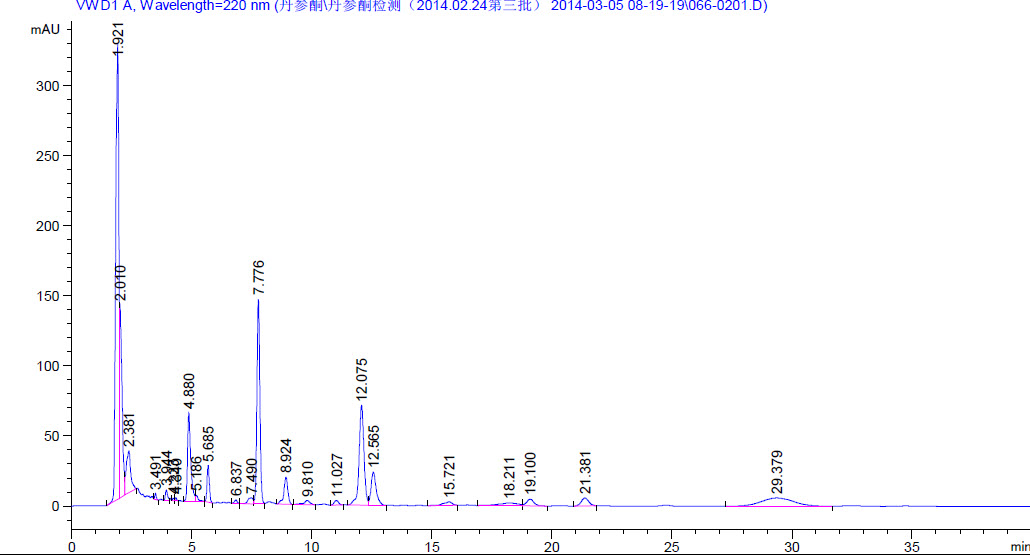


**SmWRKY1-1**

**
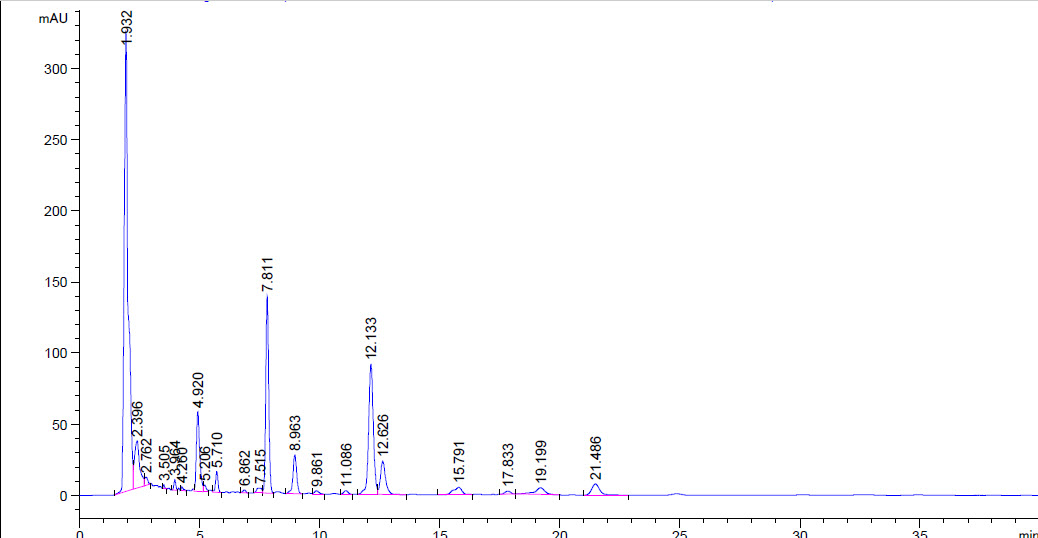
**

**SmWRKY1-2**

**
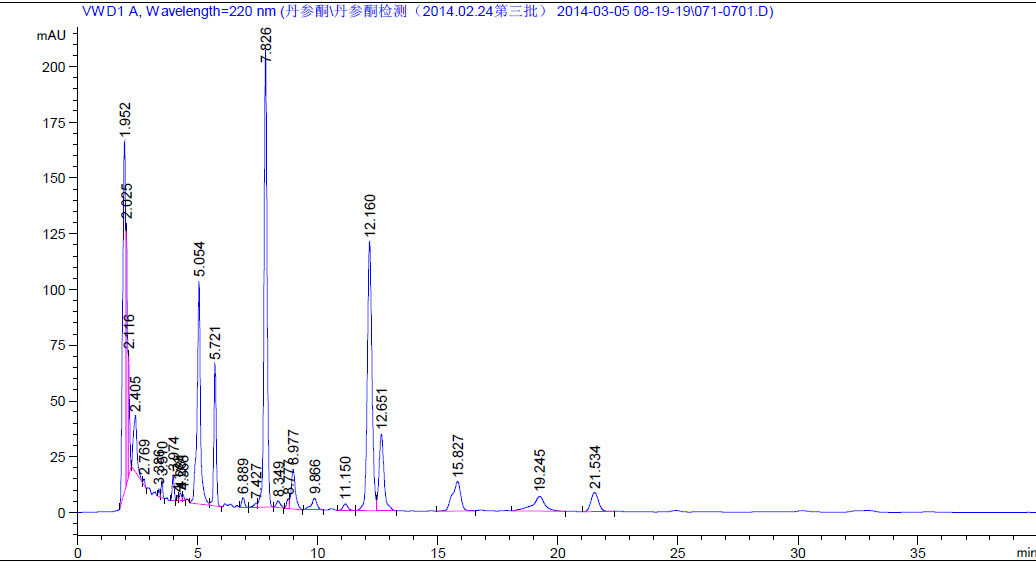
**

**SmWRKY1-3**

**Fig S3. Representative HPLC chromatograms of Empty Vector, *SmWRKY1* transgenic hairy root lines**

**.**


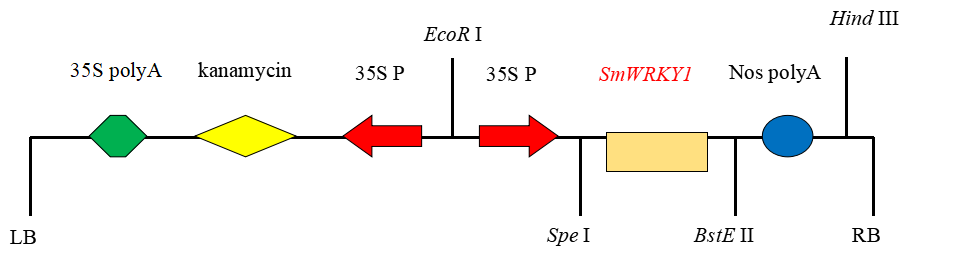


**Fig S4.** Diagrammatic sketch of *pCAMBIA2300^sm^*-*SmWRKY1.*
